# Low or oscillatory shear stress and endothelial permeability in atherosclerosis

**DOI:** 10.3389/fphys.2024.1432719

**Published:** 2024-09-09

**Authors:** Li Chen, Hua Qu, Bin Liu, Bing-Chang Chen, Zhen Yang, Da-Zhuo Shi, Ying Zhang

**Affiliations:** ^1^ Xiyuan Hospital, China Academy of Chinese Medical Sciences, Beijing, China; ^2^ National Clinical Research Center for Chinese Medicine Cardiology, Beijing, China; ^3^ NMPA Key Laboratory for Clinical Research and Evaluation of Traditional Chinese Medicine, Beijing, China; ^4^ The First Affiliated Hospital, Hainan Medical University, Haikou, China; ^5^ Graduate school, Shanxi University of Chinese Medicine, Taiyuan, China

**Keywords:** shear stress, endothelial permeability, glycocalyx, cytoskeleton arrangement, endothelial junctions, atherosclerosis

## Abstract

Endothelial shear stress is a tangential stress derived from the friction of the flowing blood on the endothelial surface of the arterial wall and is expressed in units of force/unit area (dyne/cm^2^). Branches and bends of arteries are exposed to complex blood flow patterns that generate low or oscillatory endothelial shear stress, which impairs glycocalyx integrity, cytoskeleton arrangement and endothelial junctions (adherens junctions, tight junctions, gap junctions), thus increasing endothelial permeability. The lipoproteins and inflammatory cells penetrating intima due to the increased endothelial permeability characterizes the pathological changes in early stage of atherosclerosis. Endothelial cells are critical sensors of shear stress, however, the mechanisms by which the complex shear stress regulate endothelial permeability in atherosclerosis remain unclear. In this review, we focus on the molecular mechanisms of the endothelial permeability induced by low or oscillatory shear stress, which will shed a novel sight in early stage of atherosclerosis.

## Introduction

The vascular endothelium maintains the homeostasis of the exchanges of solutes and cells between circulating blood and vascular tissues. Small molecules access to intimal layer by their concentration gradients, while larger molecules and cells pass through intimal layer via vesicles and receptors, or the injured intimal layer with higher permeability (Mundi et al., 2018). Increased endothelial permeability initiates a dysregulated transendothelial flux, leading to abnormal deposition of lipids and infiltration of inflammatory cells in the intima, which promotes the progression of atherosclerosis (Peng et al., 2019; Sedding et al., 2018; Hurtubise et al., 2016). Atherosclerosis is a major pathology of coronary artery disease, stroke, and peripheral arterial disease, which have become a principal consideration of morbidity and mortality worldwide (Libby et al., 2016; Herrington et al., 2016). Therefore, it’s crucial to elaborate the mechanisms of increased endothelial permeability in atherogenesis.

The interactions of blood flow with complex vessel geometry generate hemodynamic characteristics, including the heterogeneous spatial and temporal mechanical forces acting on the vessel wall (Zhou et al., 2014; Souilhol et al., 2020). The patterns of blood flow and the changes of hemodynamics are not consistent in the vascular system (Yamashiro and Yanagisawa, 2020). The blood flow of vertical parts in the arterial tree is usually laminar, leading to high and directed wall shear stress; while blood flow in branches and curvatures is constantly changeable and result in low or oscillatory wall shear stress (Marchio et al., 2019). Vascular endothelial cells (ECs) are constantly exposed to shear stress and respond to the changes of shear stress to regulate endothelial function. The low or oscillatory shear stress accelerates vascular dysfunction and atherogenesis, in contrast to high shear stress (Kwak et al., 2014). Chatzizisis et al. (2007) elaborated the definition of endothelial shear stress that is proportional to the product of the blood viscosity (μ) and the spatial gradient of blood velocity at the wall (endothelial shear stress = μ × dv/dy) (Chatzizisis et al., 2007). Of them, low endothelial shear stress refers to endothelial shear stress that is unidirectional at any given point but has a periodically fluctuating magnitude that results in a low time-average (approximately<10–12 dyne/cm^2^); Oscillatory endothelial shear stress is characterized by significant changes in both direction (bidirectional) and magnitude between systole and diastole, resulting in a very low time-average, usually close to 0.

Many risk factors, including hyperlipidemia, obesity, overweight, smoking, etc., are closely associated with atherogenesis. The atherosclerosis is primarily occurred near bifurcation and bends of arterial tree, where generates low or oscillatory shear stress due to disturbed blood flow (Kwak et al., 2014). The disturbed blood flow mediates the initiation of atherosclerosis by inducing vascular inflammation and excessive endothelial cell death (Souilhol et al., 2020). The initial exposure to the blood flow is arterial endothelium, which has critical role in preserving the integrity and homeostasis of blood vessel in response to hemodynamic forces and chemical signals (Davies, 2009; Heo et al., 2011). The endothelial cells are gradually changeable by low or oscillatory shear stress and then switch to atheroprone phenotype (Wang et al., 2013). Understanding the effects of low or oscillatory shear stress on endothelial cells provide mechanistic insights into the roles of complex flow patterns in increased endothelial permeability during the early stage of atherosclerosis. Therefore, this review focuses that the roles of low or oscillatory shear stress on increased endothelial permeability, which is associated with the injury of glycocalyx integrity, cytoskeleton arrangement and cell-cell junctions.

## Shear stress and glycocalyx

**FIGURE 1:**
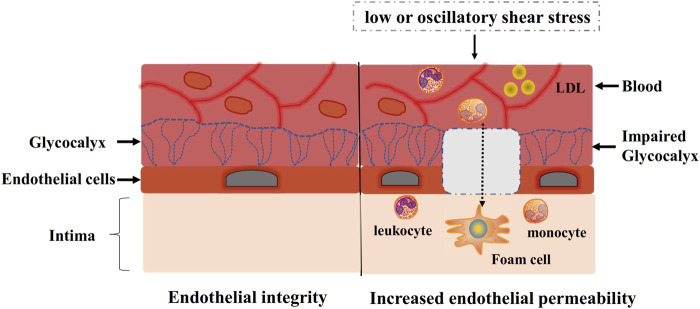
Endothelial integrity and increased endothelial permeability. Low or oscillatory shear stress impairs glycocalyx integrity, thus increasing endothelial permeability. Inflammatory cells and LDL infiltrate into intima due to increased endothelial permeability, which causes progression of atherosclerosis.

The glycocalyx is the extracellular covering and exists on the luminal surface of the plasma membrane. The thickness and structure of the glycocalyx vary from different species and is approximately 0.5–5.0 μm in humans (Alphonsus and Rodseth, 2014). The glycocalyx comprises of proteoglycans (PG), glycoproteins bound with sialic acid, glycosaminoglycans (GAG), and adherent plasma proteins (Reitsma et al., 2007). The glycocalyx acts as a negatively charged molecular sieve to maintain the integrity of endothelial barrier (Zhang et al., 2018). Moreover, glycocalyx, as a highly delicate surface structure of endothelial cells, is constantly exposed to shear stress. Low shear stress not only downregulates two major GAGs of the endothelial glycocalyx, heparan sulfate and hyaluronic acid, but also reduces the expression of PG, glycoproteins and adherent plasma proteins, thus resulting in the degradation of glycocalyx layer. Glycocalyx degradation is accompanied by increased endothelial permeability (Zhang et al., 2018) (Figure 1).

Glycocalyx integrity is dependent on blood flow patterns along the walls of the vasculature. Bai and Wang (2014) observed that the glycocalyx relocated near the edge of endothelial cells after HUVECs were subjected to 24 h low shear stress of 12 dyne/cm^2^. Following the removal of the shear stress, the glycocalyx redistributed and gradually appeared in the apical region of the cell membrane. The study showed that low shear stress decreased the distribution of sialic acid of the glycocalyx on the cell membrane, but static flow didn’t affect the distribution. The results indicated that low shear stress caused lower spatial distribution of glycocalyx layer on endothelial cell membranes compared with static flow, thus degrading glycocalyx and increasing endothelial permeability (Bai and Wang, 2014). Yao et al. (2007) examined the role of the glycocalyx in mechanotransduction by studying the well-characterized responses of endothelial cells to fluid shear stress of 15 dyne/cm^2^ for 24 h. The results showed that the glycocalyx redistribution induced by the redistribution of heparan sulfate proteoglycan from a uniform surface profile to a distinct periphery on cell surface after flow application. Moreover, endothelial cells alignment and proliferation were suppressed by removing the glycocalyx compared with normal cells after flow application, suggesting that the glycocalyx plays a pivotal role in mechanotransduction of applied shear (Yao et al., 2007). These results showed that glycocalyx played an important role in endothelial permeability when the endothelial cells exposed to different shear stress. These observations are consistent with the findings of previous studies (Giantsos-Adams et al., 2013; Koo et al., 2013) showing that glycocalyx components are synthesized at a higher rate in arteries with high shear stress compared to those with low shear stress which downregulated heparan sulfate and hyaluronic acid (Mitra et al., 2017).

Moreover, low shear stress also induces endothelial glycolysis, which is a process that releases energy by breaking down the sugar glucose and closely associated with endothelial permeability. Wu et al. (2017) found that HIF-1α activation significantly reduced the basal glycolysis and glycolytic capacity in endothelial cells subjected to low shear stress, thus increasing endothelial permeability (Wu et al., 2017). Feng et al. (2017). showed that low shear stress activates HIF 1α to increase excessive endothelial cell proliferation and permeability via nuclear factor-κB and Cezanne by the induction of glycolysis enzymes (Feng et al., 2017). Another key gene RhoA is also involved in low shear stress-induced endothelial glycolysis. Wu et al. (2021) also revealed that low shear stress induced cell activation and increased endothelial permeability by inducing endothelial glycolysis, which was associated with the activation of RhoA (Wu et al., 2021). Han et al. (2021) showed that pulsatile shear stress downregulated glycolysis in endothelial cells by KLF4-mediated epigenetic and transcriptional upregulation of GCKR expression, which increased endothelial permeability (Han et al., 2021).

## Shear stress and cytoskeleton arrangement

**FIGURE 2:**
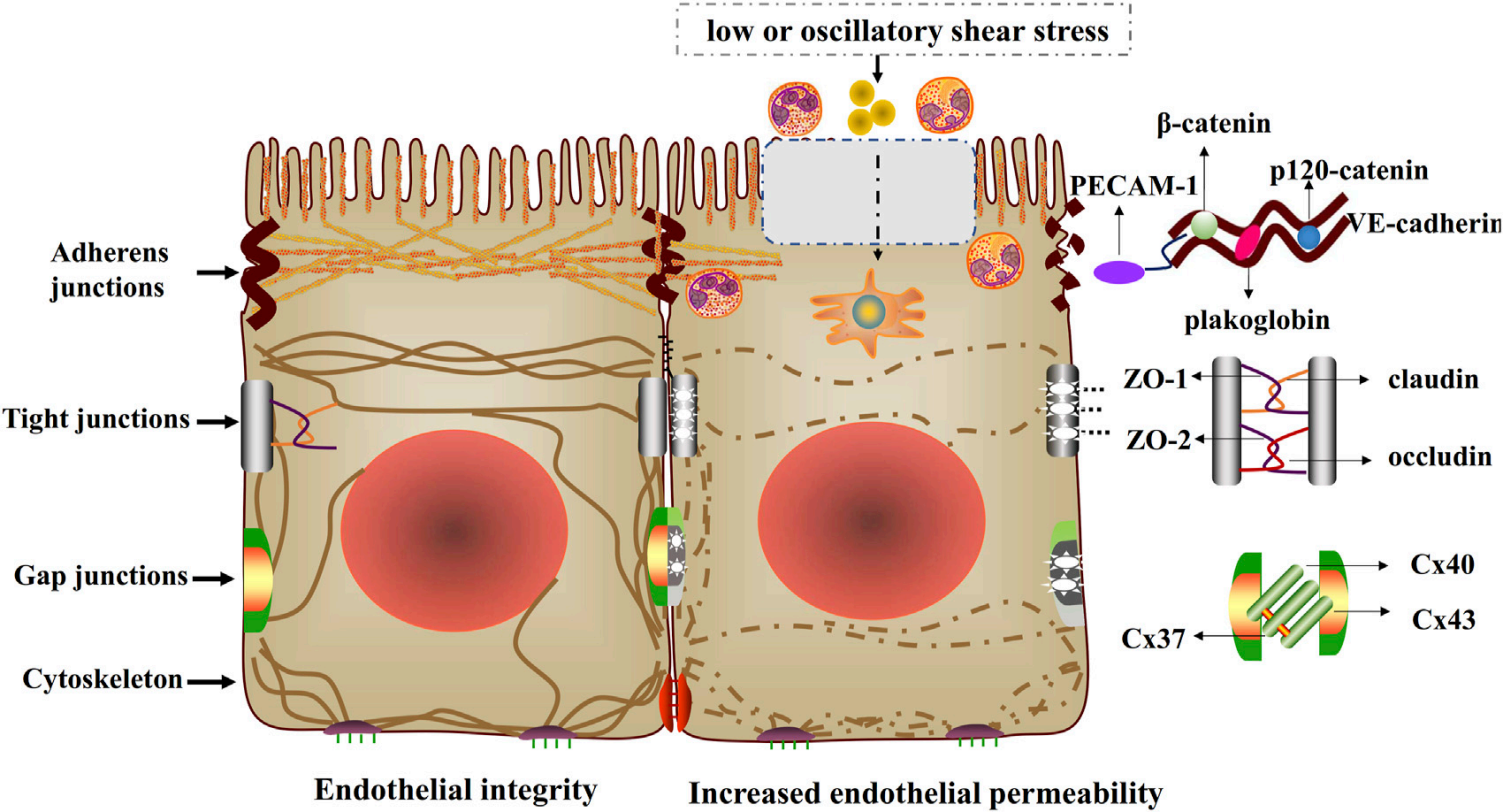
Endothelial integrity and increased endothelial permeability. Low or oscillatory shear stress impairs cell-cell junctions and cytoskeletal arrangement, thus increasing endothelial permeability. The injured cell-cell junctions include adherens junctions (VE-cadherin binds to p120-catenin, β-catenin or plakoglobin), tight junctions (occludin, claudin and ZO) and gap junctions (Cx37, Cx40 and Cx43).

The cytoskeleton has three major functions, including the spatial organization of cells’ contents, the connection of cell with the external environment, and coordinating ability to promote cells’ change and movement (Fletcher and Mullins, 2010). The cytoskeleton is a dynamic and adaptive structure whose component polymers and regulatory proteins are in constant flux (Fletcher and Mullins, 2010). Cytoskeleton arrangement is disrupted and endothelial permeability is increased when endothelial cells exposed to low or oscillatory shear stress (Figure 2).

Actin filaments, the fundamental structure of the membrane cytoskeleton, play an important role in cytoskeleton arrangement and endothelial integrity. The following studies described the critical roles of actin filaments in maintaining endothelial integrity during endothelial cells exposure to shear stress. Ting et al. (2012) found that low endothelial shear stress of 0.75 dyne/cm^2^ decreases cytoskeletal tension and disrupts distribution for actin filaments in endothelial cells, which compromises the assembly of cell-cell junctions and increases endothelial integrity (Ting et al., 2012). The mechanism is that PAK and RhoA signaling pathways are activated by low endothelial shear stress, and in turn, regulate endothelial permeability (Ting et al., 2012). Slee and Lowe-Krentz (2013) observed that fluid shear stress of 15 dyne/cm^2^ stimulates the disruptions of actin realignment in endothelial cells, which impairs VE-cadherin/β-catenin adhesions and endothelial integrity (Slee and Lowe-Krentz, 2013). The mechanism is that the phosphorylation of cofilin (an actin severing protein) decreases in the cytoplasm and increases in the nucleus, resulting in decreased correct actin realignment during endothelial cells exposed to fluid shear stress (Slee and Lowe-Krentz, 2013). Blocking stress kinases JNK and p38 reduces phospho-cofilin during fluid shear stress exposure, indicating the significance of exact actin realignment in endothelial integrity during fluid shear stress (Slee and Lowe-Krentz, 2013). Moreover, Verma et al. (2012) used stress sensitive fluorescence resonance energy transfer (FRET) sensors to measure cytoskeletal stresses in α-actinin and rearrangement of the actin cytoskeleton in endothelial cells subjected to low shear stress of 0.74 dyne/cm^2^ (Verma et al., 2012). The study showed that low shear stress decreases average actinin stress and cytoskeletal tension, which are accompanied by rearrangement of actin cytoskeleton from parallel F-actin bundles to peripheral bundles, thus increasing endothelial permeability (Verma et al., 2012). The mechanism is associated with Ca^2+^ increase and Rho GTPases signaling activation (Verma et al., 2012). These results showed that low shear stress-induced elongation and orientation of endothelial cells is due to reorganization of actin filaments and actin cytoskeleton, which accelerates an increase in endothelial permeability.

Moreover, there are some key elements involved in the regulation of shear stress in endothelial permeability. One of the key elements is transcription factor FOXC2 which controls endothelial cytoskeleton organization and thus ensures cell-cell junction stability and endothelial integrity under disturbed flow conditions. Sabine et al. (2015) found that transcription factor FOXC2 knockdown in endothelial cells subjected to oscillatory shear stress not only disrupts β-catenin link VE-cadherin to the actin cytoskeleton, but also impairs tight junctions (ZO-1) (Sabine et al., 2015). The study further demonstrated that junction stability is maintained by a FOXC2-dependent fine-tuning of the intercellular tensional cytoskeletal forces and the mechanism is associated with regulation of RhoA/ROCK/MLC signaling. These results illustrated that FOXC2 plays a critical role in maintaining endothelial integrity during oscillatory shear stress (Sabine et al., 2015). CD97, another key element, is the most broadly expressed member with roles in cell adhesion, migration and regulation of intercellular junctions. Hilbig et al. (2018) revealed that mechanical forces rapidly induce phosphorylation of CD97/ADGRE5 (pCD97) at its intracellular C-terminal PDZ-binding motif (PBM). And this phosphorylation disrupts CD97 binding to PDZ domains of the scaffold protein DLG1. Endothelial cells expressing CD97 without the PBM are more deformable, and subjected to shear stress of 1–10 dyne/cm^2^, these cells lost cell contacts and impairs actin cytoskeleton, indicating CD97 linked to cytoskeleton arrangement and affects endothelial integrity (Hilbig et al., 2018). These observations indicated that above key elements play critical role in low or oscillatory shear stress-induced increased endothelial permeability.

## Shear stress and adherens junctions

Endothelial cell-cell adherens junctions supervise fundamental vascular functions, such as controlling permeability and transmigration of circulating leukocytes, and maintaining the function of existing vessels and formation of new ones (Rudini and Dejana, 2008). The adherens junctions are particularly important for endothelial integrity and are prerequisite for the assembly of other junctional complexes. Vascular endothelial cadherin (VE-cadherin), the basic component of adherens junctions, is connected via its cytoplasmic domain to p120-catenin and β-catenin or plakoglobin (Brüser and Bogdan, 2017). Some complexes can be formed by the interaction of VE-cadherin and distinct transmembrane signaling systems, such as vascular endothelial growth factor receptor 2 (VEGFR2), vascular endothelial phosphotyrosine phosphatase (VE-PTP), and transforming growth factor (TGF) β receptor complex (Lampugnani et al., 2018). These complexes are important in regulating cell–cell junctions. Low shear stress contributes to the injury of adherens junctions, which increases endothelial permeability (Giannotta et al., 2013) (Figure 2).

Previous studies applied many advanced technologies to investigate the effects of shear stress on adherens junctions and endothelial permeability (Hur et al., 2012; Miao et al., 2005; Hofer et al., 2018). For example, Hur et al. (2012) applied 3D inter-/intracellular force microscopy technique to measure the cell-cell junctional and intracellular tensions in vascular endothelial cells monolayers under shear flow conditions. The study observed that cell-cell junctional tensions are increased by shear stress of 12 dyne/cm^2^, which are concomitant with elongated cell morphology and stress fibers, and reorganization of adherens junction proteins, such as VE-cadherin and catenins (Hur et al., 2012). This tension-mediated directional reorganization of the adherens junctions indicates that directional mechanotransduction is necessary for the ECs to regulate endothelial permeability in response to changes flow shear. Miao et al. (2005) also found that endothelial cells exposed to low shear stress (12 dyne/cm^2^) causes the reorganization of VE-cadherin in endothelial monolayers, which impairs adherens junctions (Miao et al., 2005). Hofer et al. (2018) applied an adenoviral vector with VE-cadherin-EGFP to investigate the dynamic alteration of VE-cadherin in endothelial cells subjected to low shear stress of 12 dyn/cm^2^ (Hofer et al., 2018). The results showed that cell junction remodeling during low shear stress is accompanied by VE-cadherin plaque formation, which is associated with endothelial integrity (Hofer et al., 2018).

Moreover, there are some key elements involves in shear stress-mediated increased endothelial permeability by impairing adherens junctions. Rap1, a key modulator of integrin- and cadherin-regulated processes, is indispensable for vascular stability and the formation of functional vasculature in endothelium. Lakshmikanthan et al. (2018) showed that Rap1 plays crucial roles in regulating VEGFR2-mediated angiogenesis and shear stress-induced endothelial responses (Lakshmikanthan et al., 2018). The study observed that deleting Rap1 isoform impairs *de novo* adherens junctions (VE-cadherin and β-catenin) formation and recovery from disturbed shear, indicating the important role of Rap1 in endothelial permeability (Lakshmikanthan et al., 2018). Src, a proto-oncogene, plays key roles in cell morphology, motility, proliferation, and survival. Spindel et al. (2014) found that fluid shear stress decreases Src activity and stress fiber formation in endothelial cells, whereas it increases the expression of thioredoxin-interacting protein (TXNIP) (Spindel et al., 2014). TXNIP, a biomechanical regulator, involves in the activation of Src and formation of EC stress fibers. Under low shear stress of 2 dyne/cm^2^, high expression of TXNIP increases Src Y527 phosphorylation and reduces Src activity, which impairs VE-cadherin-Src complex and F-actin stress fibers formation, thus increasing endothelial permeability (Spindel et al., 2014). Moreover, IQ domain GTPase activating protein 1 (IQGAP1) is a scaffold protein which couples cell signaling to the actin and microtubule cytoskeletons and participates in cell migration and adhesion (Rami et al., 2013). Rami et al. (2013) observed that low shear stress (1.2 dyne/m^2^) in endothelial cells induces the interaction of IQGAP1/β-catenin, which is concomitant with the impaired interactions between VE-cadherin/IQGAP1 and VE-cadherin/β-catenin. The mechanism is that IQGAP1 interacting with β-catenin causes the separation of VE-cadherin and β-catenin, thus impairing the adherent junction and endothelial integrity (Rami et al., 2013). Endothelial-to-mesenchymal transition (EndMT) is a process that low shear stress stimulates the differentiation of endothelial cells. Mahmoud et al. (2017) explored the function of Snail in low shear stress-induced EndMT, and found that exposed to low shear stress (5 dyne/cm^2^) induces Snail expression in cultured endothelial cells (Mahmoud et al., 2017). Gene silencing Snail revealed that Snail has positive regulation of EndMT markers expression (Slug, N-cadherin, α-SMA) and increases endothelial permeability to macromolecules, indicating that Snail is a necessary driver of EndMT under low shear stress and may promote early atherogenesis by enhancing vascular permeability (Mahmoud et al., 2017). Baratchi et al. (2017) showed that transient receptor potential vanilloid 4 (TRPV4) channels are expressed in preclustered structures and in a complex with β-catenin (Baratchi et al., 2017). Stimulated endothelial cells with low shear stress of 10 dyne/cm^2^ for 6 h, TRPV4 channels relocates from the basolateral membrane to basal membrane and loses the interaction with β-catenin. β-catenin is the main adapter protein of the adherens junctions at the point of cell-cell contact that is proposed to transmit the shear stress to the cell interior and cytoskeleton. These results indicated that TRPV4 might increase endothelial permeability after shear stress stimulation by relocating TRPV4 channels from adherens junctions and reducing TRPV4 with β-catenin (Baratchi et al., 2017). These results suggested that above key elements play critical roles in the impairment of adherens junctions and increased endothelial permeability due to low shear stress in endothelial cells.

Except for observing morphological changes and key molecules in adherens junctions, we also demonstrate the mechanisms of low shear stress-mediated adherens junctions damnification (Ukropec et al., 2002; Kang et al., 2014). Ukropec et al. (2002) demonstrated that the composition of the junctional complexes, such as β-catenin or α-catenin associated with VE-cadherin, are obviously decreased in endothelial cells subjected to fluid shear stress of 10 dyne/cm^2^ (Ukropec et al., 2002). The mechanism is that the impairment of α-catenin from the junctional and elevating tyrosine phosphorylation of β-catenin relates with VE-cadherin (Ukropec et al., 2002). The alteration of β-catenin phosphorylation is associated with the dissociation of protein tyrosine phosphatase SHP-2 from VE-cadherin complexes, which is mediated by low shear stress and increases endothelial permeability (Ukropec et al., 2002). Kang et al. (2014) observed that endothelial monolayers exposure to shear stress of 12 dyne/cm^2^ lowers the expression of VE-cadherin, which increases water flux and LDL leakage. However, treating endothelial cells with NO inhibitor (L-NMMA) blocks these changes, suggesting that low shear stress impairs adherens junctions and endothelial permeability by an NO dependent mechanism (Kang et al., 2014).

In addition, endothelial adherens junctions can adapt to the changes of shear stress to maintain the endothelial integrity. Noria et al. (1999) revealed that endothelial cells exposed to 15 dyne/cm^2^ of shear stress by using a parallel plate flow chamber, in order to illustrate the function of altered shear stress on the disassembly of adherens junction protein complexes (Noria et al., 1999). The results showed that shear stress contributes to partial disassembly of the adherens junction complex (α/β-catenin with VE-cadherin). Which play an important role in endothelial permeability barrier (Noria et al., 1999). Seebach et al. (2000) observed shear stress-induced change in transendothelial electrical resistance (TER) is associated with changes in cell motility and cell shape as a function of morphodynamics and accompanied by a reorganization of catenins that regulate endothelial adherens junctions. The endothelial cells exposed to shear stress of 2–50 dyne/cm^2^ and the results demonstrated that endothelial monolayers exposed to laminar shear stress respond with a shear stress-dependent regulation of permeability and a reorganization of junction-associated proteins, whereas monolayer integrity remains unaffected (Seebach et al., 2000). These different observations from above studies indicated that the endothelial monolayer has the fascinating capability to adapt accordingly to these changeable shear stress while maintaining its crucial vascular barrier function. Failure of the endothelial monolayer to adapt to changes in the magnitude or direction of forces has direct consequences on endothelial permeability (Shah and Katira, 2023), and is, therefore, regarded as an important cause of atherogenesis.

## Shear stress and tight junctions

Tight junctions play critical roles in maintaining the barrier homostasis of different compartments, and act as paracellular gates that restrict diffusion on the basis of size and charge (Zihni et al., 2016). Tight junction proteins include the claudin family that possesses barrier characteristics, and MARVEL family that conduces to barrier regulation. Besides, JAM molecules belong to tight junctions as well and regulate organization and diapedesis of the junctions (Díaz-Coránguez et al., 2019). What’s more, MAGUK family members, such as zonula occludens (ZO), contribute to the formation of scaffold, which is related with cells signaling molecules and cytoskeleton arrangement (Shen et al., 2011). Previous studies have demonstrated that the damage of tight junction proteins increase endothelial permeability under low or oscillatory shear stress conditions, which impairs the endothelial barrier function and accelerates early atherosclerotic development (DeMaio et al., 2001; Conklin et al., 2007; Conklin et al., 2002; Okano and Yoshida, 1992) (Figure 2).

Occludin is a major component of the tight junctions. Phosphorylation/dephosphorylation plays a major role in regulation of occludin and tight junctions. DeMaio et al. (2001) revealed that occludin phosphorylation increases and occludin total content decreases when endothelial cells expose to shear stress of 10 dyne/cm^2^. Alterations of phosphorylation state and total content in occludin impairs tight junction, thus increasing endothelial permeability (DeMaio et al., 2001). Conklin et al. (2002) found that low shear stress of 1.5 dyne/cm^2^ decreases occludin expression and increases endothelial cells VEGF expression, which is associated with increased endothelial permeability (Conklin et al., 2002). Moreover, Conklin et al. (2007) further found that low shear stress of 1.5 dyne/cm^2^ increases the endothelial permeability to LDL particles by using a highly controlled artery perfusion culture system (Conklin et al., 2007). The changes in endothelial permeability corresponds to an approximately 50% reduction of occludin expression, illustrating that occludin plays important roles in low shear stress-mediated increased endothelial permeability (Conklin et al., 2007). Okano and Yoshida (1992) revealed that the hip of the flow dividers of branching, a relatively low shear stress region, is covered by ellipsoidal cells with intermittent tight junctions and abnormal gap junctions in hyperlipidemic rabbits (Okano and Yoshida, 1992). The results suggested that endothelial cells exposed to relatively low shear stress may be activated, leading to increased endothelial permeability (Okano and Yoshida, 1992). These findings illustrated that occludin is not only a key player in shear stress but may also be a significant structural element in the construction of endothelial permeability within tight junctions. Except for occludin, ZO-1 is another tight junction involved in low shear stress-induced increased permeability. Besides, ZO-1 present to interact with occludin, allowing occludin to function as a seal or a gate, which would explain the increase in TER and slight reduction in cellular permeability.

Therefore, we then discussed ZO-1 in endothelial permeability-induced by low shear stress. There are plenty of advanced technologies applied to investigate the effects of different shear stress on tight junctions. Berardi and Tarbell (2009) used a hemodynamic simulator to explore the associative effects of wall shear stress and circumferential stress on EC junctions (Berardi and Tarbell, 2009). The study showed that low shear stress of 10 dyne/cm^2^ reduces the protein expression of ZO-1 and increases leaky junctions, which accelerates lipoprotein into the intima. The results indicate that shear stress impairs tight junctions stability and increases endothelial permeability (Berardi and Tarbell, 2009). Chen et al. (2019) applied a gelatin-based perfusable endothelial 3D carotid artery model to investigate the endothelial response to shear stress (Chen et al., 2019). The study observed that low shear stress (less than 0.4 dyne/cm^2^) induces the downregulation of ZO-1 levels and disorganization of F-actin cytoskeleton, which increases endothelial permeability (Chen et al., 2019). The mechanism might be associated with the increase of n ZO-1itric oxide (NO) and prostacyclin (PGI2) release, and inhibition of endothelin-1 (ET-1) release (Chen et al., 2019). Himburg et al. (2004) used a novel technique to test the interaction between fluid shear stress and endothelial permeability to macromolecules in cultured endothelial cells (Himburg et al., 2004). The results showed that low shear stress of 10 dyne/cm^2^ promotes lipoprotein infiltration into intima via an increased endothelial permeability, which might be related with increased VEGF expression and decreased occludin expression (Himburg et al., 2004).

The mechanisms of low shear stress increasing endothelial permeability by impairing tight junctions have been illustrated as followed. Rac1 has been found to be involved in a variety of cellular processes such as cell–cell or cell–substrate adhesion, apicobasal polarity, migration, invasion, and proliferation. Walsh et al. (2011) observed that 10 dyne/cm^2^ shear stress reduces membrane localization of ZO-1 and claudin-5, thus increasing endothelial permeability (Walsh et al., 2011). Treating endothelial cells with VE-cadherin inhibitor (VE-cad ΔEXD) attenuates shear-induced Rac1 activation and increased endothelial permeability (Walsh et al., 2011). The mechanism is that VE-cadherin inhibition disrupts the distribution of tight junction proteins (such as claudin-5 and ZO-1) by repressing Rac1 signaling under low shear stress (Walsh et al., 2011). Ho et al. (2019) illustrated that flow shear stress of 6 dyne/cm^2^ induces the activation of proline-rich receptor-1 (IGPR-1) compared with static flow, which modulates endothelial cells remodeling and stimulates actin stress fiber assembly by regulating ZO-1, thus increasing endothelial permeability (Ho et al., 2019). Mechanistically, shear stress activates AKT Ser/Thr kinase 1 (AKT1), resulting in phosphorylation of IGPR-1 at Ser-220. Suppressing the phosphorylation prevents low shear stress-stimulated actin fiber assembly and increased endothelial permeability. Dickkopf1 (DKK1) is a comprehensive regulator of the Wnt pathway of which activation is associated with cell proliferation and apoptosis. Li et al. (2016) found that oscillatory shear stress induces DKK1 expression and impairs endothelial tight junctions, which increases endothelial permeability and can be counteracted by siRNA knockdown or silencing DKK1 in ApoE^-/-^ mice (Li et al., 2016). The mechanism is that activating endothelial proteinase-activated receptor 1 (PAR1) and its downstream transcription factor, cAMP response element-binding protein (CREB), increases expression of DKK1 under oscillatory shear stress (Li et al., 2016). However, Gross identified a new mechanism by which endothelial cells adapt to laminar shear stress by up-regulating Sox18 to prevent the disruption of the endothelial barrier. The study demonstrated that laminar shear stress (20 dyne/cm^2^) increases the protein expression of Claudin-5 in a Sox18 dependent pattern (Gross et al., 2014). Sox18 is a transcription factor involved in endothelial barrier integrity, and knockdown of Sox18 causes the dysfunction of endothelial barrier during shear stress (Gross et al., 2014). The results indicated that laminar shear stress increases the expression of Sox18 and Claudin-5, thus protecting endothelial barrier function (Gross et al., 2014). The different results of shear stress on endothelial barrier further demonstrated the different roles of low or oscillatory shear stress on tight junctions. Low or oscillatory shear stress increasing endothelial permeability by impair tight junctions, such as decreasing occludin and ZO-1, while high laminar shear stress maintaining tight junctions by increasing Claudin-5.

## Shear stress and gap junctions

Gap junctions, integral membrane proteins, not only directly promote cytoplasmic exchange of ions, but also regulate low molecular weight metabolites of adjacent cells (Nielsen et al., 2012). Gap junctions play significant roles in physiological functions, such as propagating electrical signals and coordinating cell signaling through interacting with second messengers (Nielsen et al., 2012). Gap junction comprises of two connexons (hexamers of connexins, Cx) that align in the extracellular space (Harris, 2001). Previous studies have authenticated nearly 21 human connexin genes, such as Cx40, Cx43 and Cx37 expressed in the endothelium (Harris, 2001; Molica et al., 2014). Previous researches have illustrated that low or oscillatory shear stress increasing endothelial permeability by regulating gap junctions (Kwak et al., 2005; Gabriels and Paul, 1998; Inai et al., 2004) (Figure 2).

There is growing evidence that Cx proteins play an important role in atherosclerosis development. Cx43 is normally absent in the aortic endothelium of healthy individuals; however, it is located at branching sites of the arterial tree, which are highly susceptible to atherosclerosis development (Ramadan et al., 2012). High Cx43 expression was reported at regions of disturbed blood flow in rat aortic endothelial cells, and increased Cx43 expression was also observed in various *in vivo* studies using a model that simulates human arterial shear stress. For example, Kwak et al. (2005) showed that low shear stress of 6 dyne/cm^2^ significantly increases Cx43 expression in endothelial cells (Kwak et al., 2005). High-expressed endothelial Cx43 is at flow dividers, upstream of valves and at the shoulder of atherosclerotic plaques (Kwak et al., 2005). In such regions, the endothelium is characterized by an increased permeability and a decreased expression of endothelial nitric oxide synthase (Kwak et al., 2005). Gabriels and Paul (1998) investigate the relationship between shear stress and Cx43 expression in a segment of abdominal aorta. The study showed that Cx43 is highly localized at the downstream edge of the ostia of branching vessels and at flow dividers, regions that experience turbulent shear stress and endothelial permeability increases (Gabriels and Paul, 1998). Inai et al. (2004) revealed that Cx43 was highly localized to the sites subjected to disturbed shear from turbulent flow (Inai et al., 2004). This specific site increases Cx43 expression, and consequently supports the notion that shear stress regulate endothelial permeability by controlling Cx43 expression in endothelial cells (Inai et al., 2004). DePaola et al. (1999) used a model of controlled disturbed flows in endothelial cells to investigates the effects of low shear stress on endothelial gap junction protein Cx43. The study showed that low shear stress of 0–8.5 dyne/cm^2^ significantly increases endothelial Cx43 expression compared with no-flow controls. The results indicated that upregulation of Cx43 transcripts, sustained disorganization of Cx43 protein, and increased endothelial permeability suggest that low shear stress gradients in regions of disturbed flow regulate endothelial permeability through the expression and function of Cx43. (DePaola et al., 1999). Moreover, Xu et al. (2017) found that in blood perfused vessels, low shear stress of 1.1 or 3.2 dyne/cm^2^ acting on red blood cells (RBCs) stimulates shear dependent release of ATP, which increases EC [Ca^2+^]_i_ and endothelial gap formation, thus increasing endothelial permeability (Xu et al., 2017). These observations illustrated that high Cx43 expression at regions of low or oscillatory shear stress plays a critical role in atherogenesis by increasing endothelial permeability.

## Shear stress and PECAM-1

**FIGURE 3:**
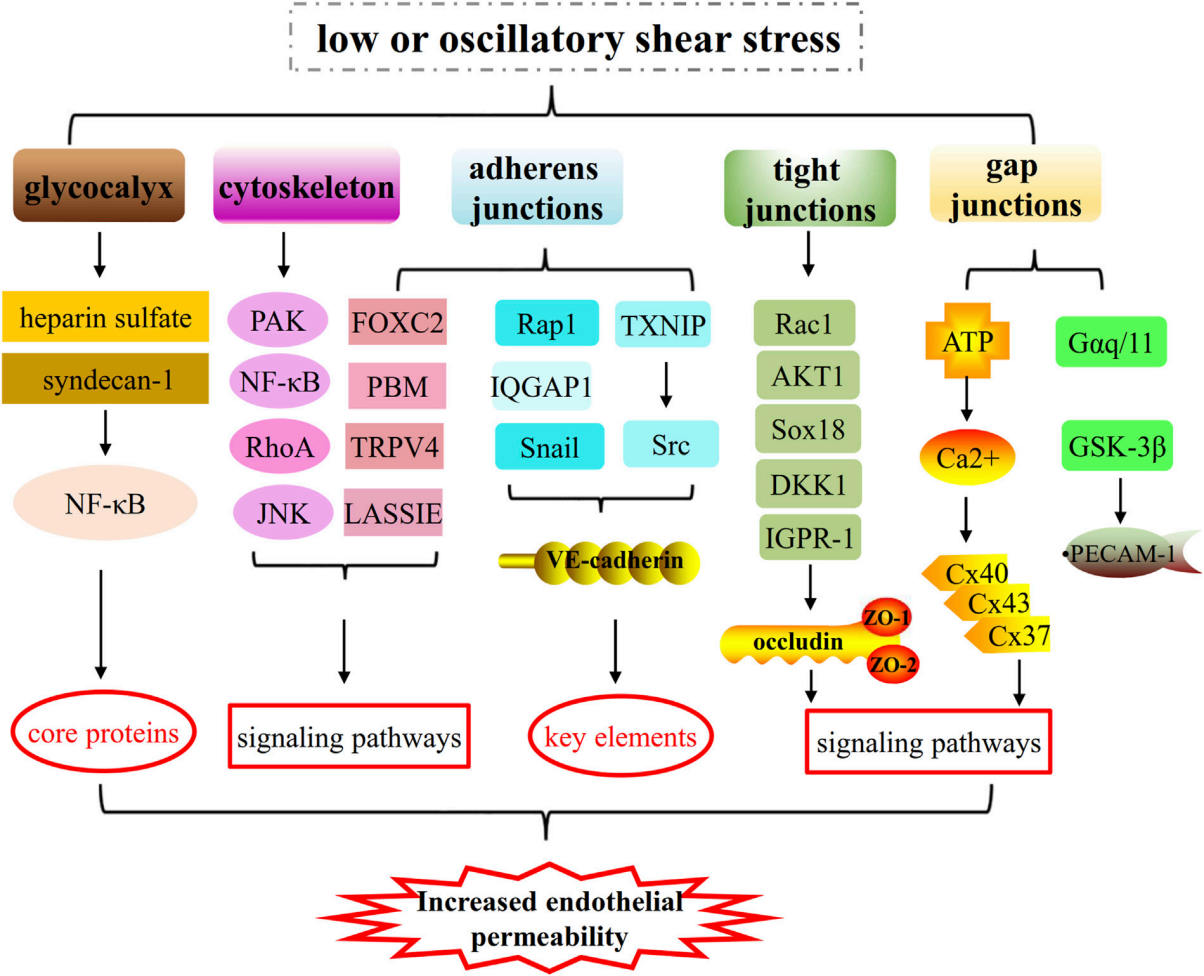
The mechanisms of low or oscillatory shear stress on increased endothelial permeability by impairing glycocalyx integrity, cytoskeletal arrangement and cell-cell junctions (such as, adherens junctions, tight junctions and gap junctions). 1) Low or oscillatory shear stress increases endothelial permeability by regulating core proteins of glycocalyx and key elements of cytoskeleton and cell-cell junctions (adherens junctions and tight junctions). 2) Low or oscillatory shear stress increases endothelial permeability by regulating PAK/NF-κB signaling, RhoA signaling, JNK signaling pathways and so on.

Platelet endothelial cell adhesion molecule (PECAM-1), a type 1 transmembrane glycoprotein of the immunoglobulin (Ig) superfamily of cell adhesion molecules, acts as vascular mechanosensor and plays an important role in angiogenesis and vascular remodeling. When endothelial cells are exposed to fluid shear stress, PECAM-1 has been shown to be tyrosine phosphorylated (Tzima et al., 2005; Lertkiatmongkol et al., 2016). Given the subcellular localization of PECAM-1 to regions of high mechanical tension, shear-induced PECAM-1 signaling may result from force-induced deformational changes in the molecule (Tzima et al., 2005), suggesting a role in sensing atheroprone hemodynamics flow. Moreover, VE-cadherin, PECAM-1 and VEGF receptor 2 form a mechanosensory complex that is in charge of shear stress sensing in endothelial cells (Conway et al., 2013) (Figures 1–3).

The following studies illustrated that PECAM-1 is a critical mediator of atherosclerosis under low shear stress. Otte et al. (2009) observed that in endothelial cells, temporal gradients in shear stress of 0–14 dyne/cm^2^ lead to a rapid dissociation of the Gαq/11–PECAM-1 complex, whereas fluid flow devoid of temporal gradients does not disrupt the complex. And fluid shear-dependent Gαq/11–PECAM-1 complex dissociation increases endothelial permeability (Otte et al., 2009). dela Paz et al. (2014) also found that Gαq/11 is rapidly dissociated from PECAM-1 followed by re-association when endothelial cells exposed to shear stress of 14 dyne/cm^2^, thus increasing endothelial permeability (dela Paz et al., 2014). Biswas et al. (2006) found that PECAM-1-null mice enhances endothelial permeability during inflammatory intervention. And in PECAM-1-null endothelial cells, β-catenin remains tyrosine phosphorylated and is accompanied by a continuous augmentation in endothelial permeability (Biswas et al., 2006). The mechanism is associated with activation of β-catenin/glycogen synthase kinase 3 (GSK-3β) signaling (Biswas et al., 2006). However, Xie et al. (2020) revealed that low shear stress of 2 dyne/cm^2^ upregulates PECAM-1 expression, leading to endothelial apoptosis and monocyte adhesion that subsequently increases endothelial permeability (Xie et al., 2020). The mechanism is that PECAM-1 siRNA transfection increases Akt and forkhead box O1 phosphorylation under low shear stress conditions. These different roles of PECAM-1-iduced by shear stress on endothelial permeability indicated that more studies are needed to investigate the exact role and mechanism of PECAM-1 in endothelial permeability under different shear stress conditions.

## Conclusion

Atherosclerosis, a progressive inflammatory disease of large and medium-sized arteries, is the main pathological changes of cardiovascular diseases (Sanz and Fayad, 2008). Vascular endothelial cells are subjected to hemodynamic forces that can activate mechanotransduction and regulate homeostasis. Pro-atherogenic low or oscillatory shear stress and atheroprotective pulsatile shear stress are two vital hemodynamic forces that modulate endothelial cells dysfunction and function (Lee and Chiu, 2019). Endothelial cells dysfunctions, including turnover enhancement, glycocalyx injury, cell-cell junction disruption, and inflammation, have been found to play vital roles in the initiation of atherosclerosis (Lee and Chiu, 2019). These responses can disrupt the intact structure of the endothelium to increase endothelial permeability and allow the penetration of lipoproteins and inflammatory monocytes to promote the progression of atherosclerosis (Wang et al., 2020; Heo et al., 2014). In this review we focus on the mechanisms that low or oscillatory shear stress increases endothelial permeability by impairing glycocalyx integrity, cytoskeleton arrangement and cell-cell junctions.

The pulsatile nature of the arterial blood flow in combination with the complex geometric configuration of the coronaries determines the endothelial shear stress patterns, which are characterized by direction and magnitude. In geometrically irregular regions, where disturbed laminar flow occurs, pulsatile flow generates low and/or oscillatory endothelial shear stress (Chatzizisis et al., 2007; Chiu and Chein, 2011; Wu et al., 2007). The low or oscillatory shear stress injures glycocalyx, cytoskeleton and cell-cell junctions, such as adherens junctions, tight junctions and gap junctions, thus leading to a leakage of the endothelial permeability barrier. The glycocalyx plays significant roles in transferring fluid shear stress to the actin cytoskeleton and initiating intracellular signaling. And cytoskeletal reorganization and biochemical responses during disturbed shear are dependent by the structural integrity of the glycocalyx and can be almost blocked if the glycocalyx is not integral (Massou et al., 2020). The interaction between cytoskeleton and cell-cell junctions is critical for tissue development and physiology, and takes part in the molecular mechanisms, such as regulating cell shape, motility and growth (Charras and Yap, 2018). Low or oscillatory shear stress injures the function of the actin cytoskeleton in the organization and function of cell–cell junctions, thus increasing endothelial permeability, while high shear stress might play a beneficial role. KLF2 are core shear-stress dependent transcription factors which regulate cell-cell junctions, and Huang et al. (2017) showed that high shear stress induces KLF2 which act to promote adherens and tight junctions. When the glycocalyx is damaged by low or oscillatory shear stress, the subsequent dysfunctions of cytoskeleton and cell-cell junctions will also occur, forming a malignant circle, thus increasing endothelial permeability and accelerating atherosclerotic development.

The vascular barrier is essential for normal tissue homeostasis. The barrier is formed by tight junctions, adherens junctions and gap junctions that join endothelial cells, with additional contributions from the endothelial cell glycocalyx and cell cytoskeleton on the luminal surface and basement membrane and mural cells on the abluminal surface (Wettschureck et al., 2019). Barrier disruption is accompanied by an increase in endothelial permeability, which occurs in early stages of atherogenesis. These pathological findings are important when considering therapeutic strategies for treating early stage of atherosclerosis. Preventing the entry and subsequent intimal retention of ApoB-containing lipoprotein, particularly at an early stage of atherosclerosis, are critical in the prevention of atherosclerosis. For example, statins lower the plasma concentration of lipoproteins and can reduce the entry of lipoproteins in the intima (Nakashima et al., 2008). Targeting of inflammatory pathways also provide an additional treatment in early stage of atherosclerosis. Dexamethasone (DXM), an anti-inflammatory steroid drug, can inhibit atherosclerosis development via decreasing intimal macrophage recruitment and foam cell formation (Zhang et al., 2017).

Considering the critical roles of low or oscillatory shear stress in endothelial permeability during early stage of atherogenesis, some problems need to be solved urgently. Many unknown knowledge still needs to be uncovered to develop endothelial barrier-targeted therapies in atherosclerosis (Peruzzi et al., 2018). Especially, applying the therapeutic strategies to promote normal shear stress in the branches and bends of arteries, which may contribute to maintain glycocalyx integrity, cytoskeleton arrangement and cell-cell junctions (Peruzzi et al., 2018; Tarbell and Cancel, 2016). First, as novel potential therapeutic targets are increasingly distinguished, including EndoMT reversal, the assumption to treat atherosclerosis via above targets might be proposed and ultimately applied in human trial (Li et al., 2018). Second, studies have showed that low shear stress (3–6 dyne/cm^2^) increases PCSK9 expression, and lipid-induced increase in PCSK9 expression is higher at low shear stress (Yurtseven et al., 2020; Ason et al., 2014). PCSK9 inhibitors might contribute to improve endothelial dysfunction-induced by low shear stress. Moreover, a principal challenge in the region is the integration of a large quantity of data on endothelial cells subjected to disturbed shear flow at the molecular level, and then to identify the shear stress-specific endothelial gene expression and phenotype (Monfoulet et al., 2017). Therefore, understanding the mechanisms of low or oscillatory shear stress in increased endothelial permeability promotes to find new diagnostic markers and develop novel therapies.
